# Numerical Investigation of Fine Particulate Matter Aggregation and Removal by Water Spray Using Swirling Gas Flow

**DOI:** 10.3390/ijerph192316129

**Published:** 2022-12-02

**Authors:** Jianghai Qian, Junfeng Wang, Hailong Liu, Haojie Xu

**Affiliations:** 1 Department of Fluid Mechanics, School of Energy and Power Engineering, Jiangsu University, Zhenjiang 212013, China; 2 Department of Fluid Machinery, School of Energy and Power Engineering, Jiangsu University, Zhenjiang 212013, China; 3 Department of Engineering Thermal Physics, School of Energy and Power Engineering, Jiangsu University, Zhenjiang 212013, China

**Keywords:** swirling flow field, Euler’s multiphase flow model, fine particulate matter, population balance model, turbulent aggregation

## Abstract

In this paper, a mathematical model based on the two-fluid frame model coupled with the population balance model which considers the aggregation of particles and droplets in detail for cyclonic spray dedusting is proposed. The model is applied to study the characteristics of multiphase flow field and the effects of the gas velocity, spray volume, and particle concentration on the removal efficiency. In addition, the simulation results are verified by the experimental data. The results suggest that the turbulence kinetic energy increases near the wall as the inlet velocity increases, and the spray region increases as the spray volume increases. This is conducive to turbulent mixing of the particles and droplets, and the agglomeration efficiency of the particles is improved, so the particle size increases, and the particle removal efficiency increases to 99.7% by simulation results are within the allowable range of error (about 99–99.5% in dedusting efficiency by experimental data). As the particle concentration increases, the particle removal efficiency initially increases, then decreases and reaches the highest value at 2 g/m^3^, which is due to the limited adsorption efficiency of the spray droplets. The results are helpful for providing a theoretical basis for spray to promote agglomeration of particles and improving the dust removal efficiency in the swirl field.

## 1. Introduction

Fine particulate matter (FPM) released from coal combustion is the main pollutant in the environment. The suspension of FPM (mainly PM2.5, i.e., particle size smaller than 2.5 microns) in the atmosphere always poses serious hazards to human health and the environment. Due to its small size, it can easily travel deep inside the respiratory tract, causing respiratory diseases and even lung cancer. Although the existing dust removal devices have removal efficiencies of as high as 99% or more, they still fall short of catching very fine particulate matter, and a great deal of FPM is still observed in the atmosphere [1,2,3]. Therefore, the study of FPM removal technology is particularly important.

In industrial applications, different dust removal systems are currently applied to reduce PM emission. Available dust removal technologies vary in removal efficiency, collected PM size and costs [4]. Fabric filters and electrostatic precipitators (ESPs) have the highest removal efficiency for PM2.5. Fabric filters are mainly based on the sieve effect, produced by filtering textiles on which particles are captured. However, they have high maintenance costs due to the rapid clogging of the filter, which can cause re-suspension of particles previously collected [5]. ESPs remove FPM from the flue gas by the electric force, which also have high investment and operational costs [6]. Higher costs make fabric filters and ESPs economically suitable only in industrial application. Wet scrubbers have some advantages over fabric filters and ESPs: scrubbers are simpler and have lower capital and maintenance costs. Collection efficiency of wet scrubbers reaches over 80% of FPM with design optimization [7]. However, one of the main drawbacks of wet scrubbers is the high amount of water needed for particle removal [8]. As an alternative, cyclonic separators as well as other inertial separation systems have been widely used, which also have low installation, operation and maintenance costs [9]. Nevertheless, cyclones’ collection efficiency generally only reaches values between 60% and 80% for particle diameters between 2 and 10 μm, making them a good choice for a pre-collection device; moreover, they can be attached to other equipment with higher efficiency, depending on the process requirements [10].

Innovation methods to improve the collection efficiency of a conventional cyclone have been investigated. Spray in the interior of the cyclone can promote the agglomeration of particles, which has been regarded as an effective and inexpensive method to deal with FPM [11]. In this device, the strong cyclonic flow, also called swirling flow, is introduced to increase the relative velocity of the dust particles and droplets [12], enhance the gas–liquid turbulence, increase the contact probability between the fine particles and droplets, and accelerate the aggregation and growth of the fine particles [13]. Bo W et al. [14] innovated a new fine particle removal technology—Cloud-Air-Purifying—which aggregates FPM and increases the particle size and found that the collection efficiency of FPM was improved compared to traditional gas cyclone. Luke S. Lebel et al. [15] discussed the washing mechanism of cyclone spray scrubber and the numerical model was established to predict the aerosol effectively. Krames and Buttner [16] found that cyclone scrubber was more economical and feasible than a wet scrubber in cleaning. For particles larger than 3 μm, the collection efficiency reached 99%, and the water consumption was 0.05–0.25 L/m^3^. Lee et al. [17,18] performed both experimental and theoretical research on the particulate scrubbing efficiency based on the aerodynamic diameter of the particles to study the development and application of a novel swirl cyclone scrubber. They derived a model of the particle collection efficiency due to Brownian diffusion, inertial collisions, and gravitational sedimentation. Ali et al. [8,19] investigated a model of a centrifugal wet scrubber via numerical simulations and found droplet carryout has an important effect on the predicted collection efficiency. Liu et al. [20] proposed a tangential swirl coagulating device and found that swirling flow is beneficial to the mixing and collision of fine particles. A survey of existing research revealed that the enhancing effect of cyclonic spray dedusting on the efficiency has been demonstrated, but the influence of the swirl motion on the multiphase flow characteristics and removal efficiency has not yet been quantitatively analyzed.

With the reduction in computing costs in recent years, numerical simulations have been extensively adopted for both scientific study and engineering design. Wang et al. [21] carried out a study on a spray scrubber using the discrete phase model (DPM) to simulate urea particle removal, and they predicted the removal efficiencies under different conditions. However, the DPM requires a great deal of computing resources and cannot provide information such as the collision and coalescence effects of the particles. Widespread theoretical research is being conducted on the population balance model (PBM) based on the two-fluid frame. It is used to describe the spatiotemporal evolution of the particle size distribution (PSD) of the dust particles and water droplets. Duangkhamchan et al. [22] developed a multi-flow model combined with the PBM as an alternative approach for modelling the spray in a tapered fluidized bed coater and predicted the temporal evolutions of the distributions with respect to the particle size and the liquid distribution. Akbari [23] studied the segregation of a wide range of PSDs in an industrial gas phase polymerization reactor using a computational fluid dynamics (CFD)-PBM coupled model, which helped to reveal the physical details of the multi-phase flow field. As was previously mentioned, few numerical studies have investigated the dynamic properties and interaction mechanism of water droplets and aerosol particles during the dedusting process, and the PBM has not been applied to cyclone spray dedusting.

In this study, a mathematical model based on the two-fluid frame model coupled with the population balance model for cyclone spray dedusting was developed, which considers the aggregation of the particles and droplets in detail. The model was applied to study the multiphase flow characteristics and the key factors affecting the particle removal efficiency, such as the gas flow velocity, spray flow rate, and particle concentration. The results of the CFD simulation are helpful for providing a theoretical basis for spray to promote agglomeration of particles and improving the dust removal efficiency in the swirl field, which also can provide the guidance for optimum design of a cyclonic spray scrubber in practical engineering applications.

## 2. Numerical Simulations

### 2.1. Mathematical Model Two-Fluid (Euler–Euler) Model

The particle size of dust and droplets is very small, and they are sparsely distributed in space; however, the interaction between the particles should be taken into account. Therefore, the two fluid (TF) model (primary phase is the air and the secondary phase is the particle) is used to calculate the velocity field [24].

The equations for the conservation of mass and momentum can be written as follows:

(1) $$\frac{\partial }{\partial t}\left(\varphi \rho \right)+\nabla \cdot \left(\varphi \rho v\right)=0,$$

(2) $$\frac{\partial }{\partial t}\left(\varphi \rho v\right)+\nabla \cdot \left(\varphi \rho vv\right)=-\varphi \nabla p+\nabla \cdot \bar{\tau }+\varphi \rho g+\sum_{p=1}^{2}R_{p}+\left(F+F_{vm}\right)$$

where φ is the volume fraction of each phase, ρ is the phase density, *v* is the phase velocity, ∇p is the pressure gradient, $\bar{\tau }$ is the stress tensor, φρg is the gravity term, g is the acceleration due to gravity,$\;\overset{2}{\underset{p=1}{\sum }}R_{p}$ is the interphase drag term, *F* is the additional physical force, and $F_{vm}$ is the virtual mass force.

The drag force $R_{p}$ uses a simple interaction term of the following form:

(3) $$\overset{2}{\underset{p=1}{\sum }}R_{p}=K_{p}\left(v_{p}-v\right)$$

where $K_{p}$ is the interphase momentum exchange coefficient that can be written in the following general form:

(4) $$K_{p}=\frac{\rho_{p}f}{6\tau_{p}}d_{p}A_{i}$$

where $A_{i}$ is the interfacial area, $\tau_{p}$ is the particulate relaxation time, $d_{p}$ is the diameter of the particle or droplets. The Schiller and Naumann model is acceptable for general use for all fluid–fluid pairs of phases, which can be written in the following form:

(5) $$f=\frac{C_{D}R_{e}}{24}$$

The components of the multiphase flow in this paper are air, dust particles, and water mist, and turbulence plays an important role in the aggregation of the particles. Compared with the standard k−ε model, the Reynolds stress model (RSM) turbulence model introduces the correlation terms of the rotation and curvature to avoid the occurrence of a negative normal stress, so the RSM turbulence model was applied [25]. The Reynolds stress term includes the turbulence closure, which must be modelled to solve Equation (2).

In the RSM, the transport equation can be written as follows:

(6) $$\frac{\partial }{\partial t}\left(\rho \bar{u_{i}^{\prime }u_{j}^{\prime }}\right)+C_{ij}=P_{ij}+D_{ij}+\emptyset_{ij}+\varepsilon_{ij}$$

The turbulent diffusion term is

(7) $$D_{T,\;ij}=-\frac{\partial }{\partial x_{k}}\left[\rho \bar{u_{i}^{\prime }u_{j}^{\prime }u_{k}^{\prime }}+\bar{p^{\prime }\left(\delta_{kj}u_{i}^{\prime }+\delta_{ik}u_{j}^{\prime }\right)}\right]$$

The stress production term is

(8) $$P_{ij}=-\rho \left(\bar{u_{i}^{\prime }u_{k}^{\prime }}\frac{\partial \bar{u_{j}}}{\partial x_{k}}+\bar{u_{j}^{\prime }u_{k}^{\prime }}\frac{\partial \bar{u_{i}}}{\partial x_{k}}\right)$$

The pressure strain term is

(9) $$\emptyset_{ij}=p^{\prime }\left(\frac{\partial u_{i}^{\prime }}{\partial x_{j}}+\frac{\partial u_{j}^{\prime }}{\partial x_{i}}\right).$$

The dissipation term is

(10) $$\varepsilon_{ij}=-2\mu \frac{\partial u_{i}^{\prime }}{\partial x_{k}}\frac{\partial u_{j}^{\prime }}{\partial x_{k}}$$

In Equations (3)–(7), $\delta_{ij}$ is the Kronecker factor, and $\mu_{t}$ is the molecular viscosity.

### 2.2. Population Balance Model

The population balance equation (PBE) model is used to calculate the aggregation effect of the particles in this paper, and the phenomenon of the breakup of particles is not considered. Based on the particle sparsity hypothesis, the zero-dimensional equilibrium equation of the PSD function in the Eulerian coordinate system with only particle aggregation considered can be written as follows [26]:

(11) $$\begin{array}{rr} \frac{\partial n\left(v,t\right)}{\partial t}= & \frac{1}{2}\overset{v}{\underset{V_{min}}{\int }}\beta \left(v-u,u,t\right)n\left(v-u,t\right)n\left(u,t\right)du\\ & -n\left(v,t\right)\overset{v_{max}}{\underset{v_{min}}{\int }}\beta \left(v,u,t\right)n\left(u,t\right)du, \end{array}$$

where n(v,t) denotes the number density function of the particles with volume *v* at time *t* (1/m^3^), and β(v−u,u,t) denotes the aggregation nucleus of the particles with volume *u* and *v* − *u* (m^3^/s). The first term on the right side of the equation denotes the number of new particles with volume *v* generated through aggregation, and 1/2 indicates that two particles participate simultaneously in a single aggregation event. The second term de−notes the number of particles whose volume vanishes as v as a result of aggregation into larger particles.

### 2.3. Aggregation Kernel Model

In practical engineering applications, the droplet size produced by various atomizers is larger than 10 μm [27], so there is no aggregation caused by the Brownian motion of the droplet particles. Although the aggregation of dust particles occurs when the local humidity increases to a certain value, the Brownian aggregation of <1 μm dust particles can still be neglected compared with the capture of dust by droplets. Therefore, the free molecular aggregation model is not discussed in this paper. The turbulent aggregation model is selected for use in this paper.

In the turbulent flow field, the turbulence within the fluid always generates eddies, which in turn dissipate the energy. Energy is transferred through the largest eddies to the smallest eddies where it is dissipated via viscous interaction. The size of the smallest eddies is the Kolmogorov microscale, which is expressed as a function of the kinematic viscosity v and the turbulent energy dissipation rate ε as follows:

(12) η=(v3ε)14.

According to the sizes of the two particles, aggregation can occur, and this is described in terms of the following three models.

(a) When the diameters of two particles *i* and *j* are $d_{i}<\eta$ and $d_{j}<\eta$, based on the study conducted by Saffman and Turner [28], the collision rate is expressed as follows:

(13) $$\beta \left(d_{i},d_{j}\right)=\zeta_{T}\sqrt{\frac{8\pi }{15}}\gamma \frac{{\left(d_{i}+d_{j}\right)}^{3}}{8}$$

where $\zeta_{T}$ is a pre-factor that takes into account the capture efficiency coefficient of the turbulent collision. The expression of the empirical capture efficiency coefficient of turbulent collision can be written as [29]:

(14) $$\zeta_{T}=0.732{\left(\frac{5}{N_{T}}\right)}^{0.242}\;(N_{T}\geq 5)$$

where $N_{T}$ is the ratio between the viscous force and the Van der Waals force:

(15) $$N_{T}=\frac{6\pi \mu {\left(d_{i}+d_{j}\right)}^{3}\dot{\lambda }}{8H}$$

where *H* is the Hamaker constant, a function of the particle material; $\dot{\lambda }$ denotes the deformation rate:

(16) $$\dot{\lambda }={\left(\frac{4\varepsilon }{15\pi v}\right)}^{0.5}$$

 γ is the shear rate, and it can be written as

(17) $$\gamma =\frac{\varepsilon^{0.5}}{v}$$

(b) When the diameters of the two particles *i* and *j* are $d_{i}>\eta$ and $d_{j}>\eta$, the aggregation rate can be expressed using Abrahamson’s model [30]:

(18) $$\beta \left(d_{i},d_{j}\right)=\zeta_{T}2^{3/2}\sqrt{\pi }\frac{{\left(d_{i}+d_{j}\right)}^{2}}{4}\sqrt{\left(U_{i}^{2}+U_{j}^{2}\right)}$$

(c) When the diameters of the two particles *i* and *j* are $d_{i}\geq \eta \;\mathrm{and}\;d_{j}<\eta$ or $d_{i}<\eta \;\mathrm{and}\;d_{j}\geq \eta$, Zheng’s model [31] can be used to express the aggregation rate:

(19) $$\beta_{t}\left(d_{i},d_{j}\right)=\sqrt{\frac{8\pi }{15}}\sqrt{\gamma }\frac{{\left(d_{i}+d_{j}\right)}^{3}}{8}{\left(\frac{2\eta }{d_{i}+d_{j}}\right)}^{0.08+0.897St},$$

where St is particle relaxation time scale and the fluid characteristic time scale ratio; $U_{i}^{2}\;$and $U_{j}^{2}\;$are the mean square velocities of particles *i* and *j*, respectively.

### 2.4. Boundary Conditions and Computational Method

The simulated flue gas at the inlet is set as a two-phase flow with a particle phase and gas phase. The PBM model is introduced in the Fluent software. During the computations, the inlet and outlet are set as the velocity inlet and pressure outlet, respectively. The gas flow velocity in the inlet varies from 8 to 16 m/s. The flow rate of the nozzle is set as 1.2–2.4 L/min. The dust loading is maintained at 1–3 g/m^3^. According to the experimental research on pressure swirl spraying, including particle size analysis, the particle size distribution of each phase is listed in Table 1.

A pressure-based solver is used in the CFD calculations, a coupling algorithm is used for the pressure–velocity coupling term, a second order upwind difference scheme is used for the momentum discretization term, and a first-order scheme is used for the turbulent kinetic energy and turbulent dissipation rate. The transient simulation is carried out using a time step of 0.0001 s. The convergence criterion of all of the scalars requires that the normalized residuals be less than 10^–4^. The near-wall treatment includes the non-equilibrium wall function. Due to the specific requirements regarding the PSD and the respective volume fraction ratios, the more advantageous inhomogeneous discrete method is adopted.

## 3. Results and Discussion

### 3.1. Model Validation

**Figure 1:**
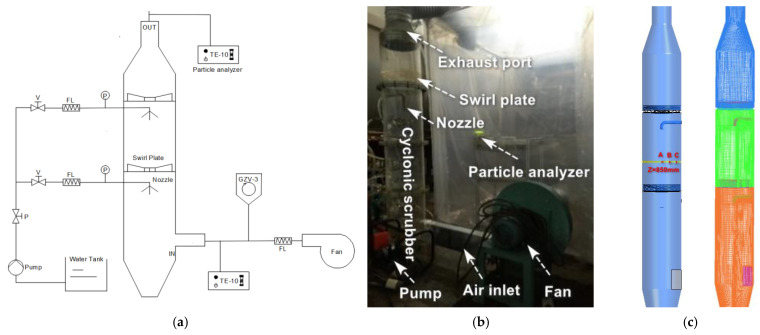
Cyclonic spray scrubber system: (**a**) Schematic diagram; (**b**) Experimental field setup; and (**c**) Geometric model (measure points A, B and C) and Computational grid.

A schematic diagram of the experimental setup is presented in Figure 1a. Contaminated gases were introduced via tangential inlets at the bottom of the cyclone, and the rotation of these gases in the body of the system induced a vortex. Some large particles were thrown to the wall for separation under the action of centrifugal force. Fine particles were not collected with the gases rotated upward along the wall of the cyclone, and while the gases migrated through the system, the water spray provided a counter-current flow arrangement that cleans the gases by washing out the suspended aerosols. A swirl plate was placed above the gas inlet to enhance the mass transfer through the scrubber. Finally, the purified flue gas was discharged from the top to achieve efficient removal of fine particles. The field experimental device of the cyclonic spray scrubber was established as shown in Figure 1b. The cyclonic spray scrubber has a rectangular tangential inlet with 0.1 m height and 0.05 m width and consists of a cylindrical Perspex column (2 m in height and 0.2 m in diameter) with a spray nozzle at the center of the scrubber. 

In this stage, the Fluent software (version 19.2, 1987–2018 ANSYS, Canonsburg, PA, USA) was used to simulate this experimental model. The Euler two-fluid model and population balance model described above were adopted. All of the boundary conditions were set according to the experimental data. The PSD according to the experimental data was divided into seven bins (Table 1). According to the cyclonic spray scrubber in the experimental system, a simplified three-dimensional numerical model was constructed (Figure 1c). The domain containing 2302,306 cells was discretized using a structured hexahedral mesh in the ANSYS ICEM software (Figure 1c). The value of minimum orthogonal quality was equal to 0.15, which indicated that the mesh quality could meet the calculation requirements. We also tested three grid domains in our preliminary computation containing 1,801,185, 2,302,306, and 2,875,240, respectively. The mesh sensitivity test was conducted to prove that when the mesh density was 2,302,306 cells, mesh independence was achieved since a further increase in the cell number only caused a 2% change in the predicted airflow velocity.

In addition to the mesh independence study, the base model results were validated against field measurement data before accepting the model for use in parametric studies. In this study, gas velocities and dust concentration were employed for the base model validation. We used a high precision anemometer to measure the airflow at three points (A, B, and C in Figure 1c) and a TE-10-800 Anderson particle detector to measure the particle concentration at the outlet. Table 2 compares the model-estimated and measured air flow velocities and dust concentrations. Considering the influence of the measurement error, the simulation results are acceptable. In general, the model developed in this study has a high accuracy and application value in calculating the dedusting efficiency.

### 3.2. Gas Velocity Distribution in a Cyclonic Spray Scrubber

**Figure 2:**
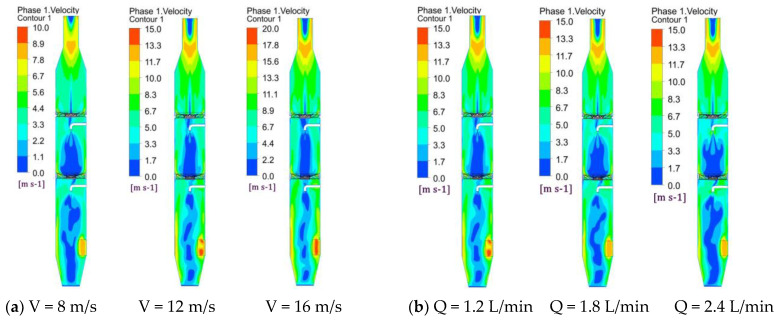
Cloud diagram of the airflow velocity distribution in section Y = 0 for (**a**) different gas velocities and (**b**) different spray volumes.

Figure 2 shows a cloud diagram of the gas flow velocity distribution in section Y = 0. The color gradient in Figure 2 indicates the magnitude of the gas velocity. As can be seen from Figure 2 the velocity distribution within the scrubber is basically axisymmetric. Due to the effect of the centrifugal force on the airflow and the effect of the blade guide, the velocity is the smallest in the central area below the nozzle. It gradually increases along the radial direction from the center to the wall, and gradually decreases to zero near the wall boundary layer. The flue gas cannot flow upward through the blind plate, which is located at the center of the swirl plate, so the airflow velocity is very small in the center of the cyclonic spray scrubber. As the flow velocity increases, the central spray region is strongly affected by the vortex shear, and the flow velocity increases slightly. As the spray flow rate increases, the flow velocity of the flue gas in the spray area decreases sharply, and the low airflow velocity area affected by the spray becomes larger. This is mainly due to the higher concentration and speed of the droplets due to the larger nozzle flow rate, and the air flow is more affected by the droplet resistance, so the air flow decreases rapidly.

### 3.3. Droplet Distribution

**Figure 3:**
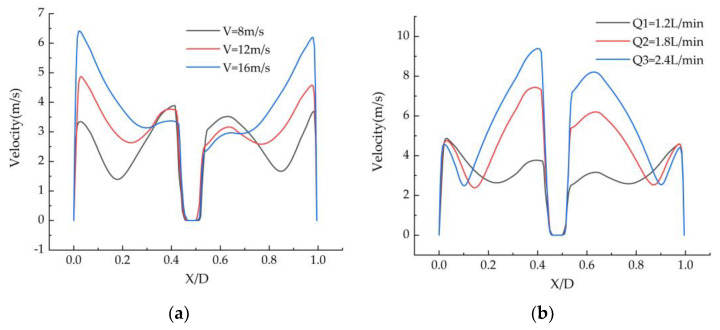
Distribution of the droplet velocity at Z = 850 mm for (**a**) different gas velocities and (**b**) different spray volumes.

Figure 3 shows the velocity distribution of the droplets at transverse Z = 850 mm under different operating conditions. As can be seen from Figure 3, as the gas velocity increases, the droplet velocity decreases slightly in the region of 0.3 < X/D < 0.7, while the velocity in the regions on both sides increases. As the spray volume increases, the droplet velocity increases in the region of 0.1 < X/D < 0.9, while the velocity decreases slightly in the regions on both sides. It can be seen from the characteristics of the swirl flow field that the airflow velocity in the central area is small, and the airflow is obviously affected by the droplets. The larger the flow rate, the larger the area of the droplets along the ra−dial direction. The airflow velocity on both sides is large, and the droplet is obviously affected by the airflow. The larger the velocity, the stronger the droplet carried by the airflow.

**Figure 4:**
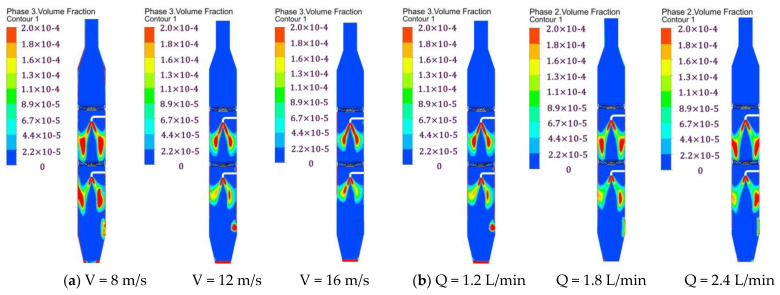
Cloud diagram of the distribution of the volume fraction of the droplets in section Y = 0 for (**a**) different gas velocities and (**b**) different spray volumes.

Figure 4 presents a cloud diagram of the volume fraction distribution of the droplets in section Y = 0. As can be seen from Figure 4, the distribution of the droplet volume concentration is hollow and conical. The central area is low, and the side wall area is high. The droplet covers the interaction area between the flow passage section and the fine particles, which is conducive to the collision and agglomeration of the droplets and fine particles. The distribution of the droplet particle volume concentration is basically the same under different inlet gas velocities. As the inlet velocity increases, the axial velocity of the droplets in the central region decreases gradually under the action of the gas–liquid two-phase velocity difference, and the droplet movement distance along the axial direction becomes shorter. However, the centrifugal force on the droplet in the side wall region increases gradually, and the droplet concentration along the radial direction decreases gradually. 

### 3.4. Particle Size Distribution under Different Conditions

**Figure 5:**
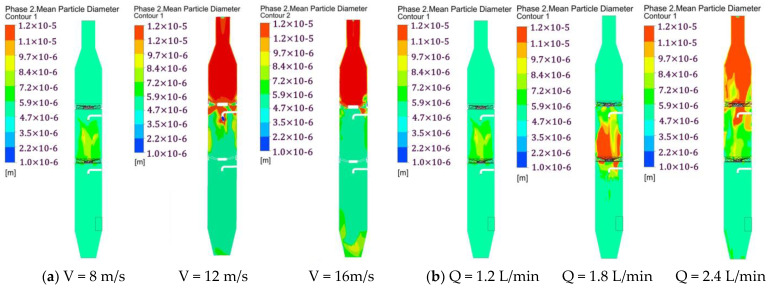
Particle size distribution under different conditions in section Y = 0 for (**a**) different gas velocities and (**b**) different spray volumes.

The average particle size distribution can be calculated using the population balance model. Figure 5 shows the average particle size distribution under different conditions. It can be seen from Figure 5 that the particle size in the spray area is large when the airflow velocity is small. As the flow velocity increases, the particle size gradually increases and tends to become stable after passing through the swirl plate. This is mainly because the air around the nozzle changes the direction of the velocity due to the influence of the droplets, and a reflux area is generated under the nozzle, which causes the flue gas and droplets to be sucked up, increases the interaction time between the particles and droplets, and enhances the agglomeration effect. As the flow velocity increases, the turbulent kinetic energy increases, the collision between the droplets and particles through the swirl plate becomes more severe, the particles significantly agglomerate, and the particle size along the axial direction gradually increases.

Figure 5b shows the particle size distribution under different spray volume flow rates in section Y = 0. Figure 5b shows that the particle size gradually increases along the axial direction as the amount of spray increases. As the nozzle flow rate increases, the concentration and velocity of the droplets increase, the gas–liquid turbulence becomes more intense in the spray area, the airflow speed decreases rapidly due to the effect of droplet resistance, and the tiny particles with an airflow movement speed are also reduced. The interaction time between the particles and droplets increases, and the interparticle collision coalescence strengthens, so the particle size significantly increases.

### 3.5. Particle Removal Efficiency under Different Operating Conditions

#### 3.5.1. Effect of Gas Flow Velocity on Particle Removal Efficiency

**Figure 6:**
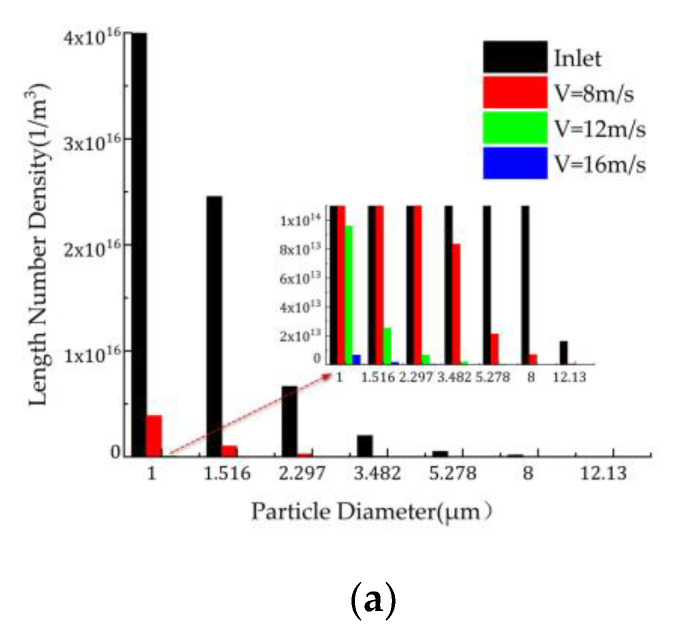

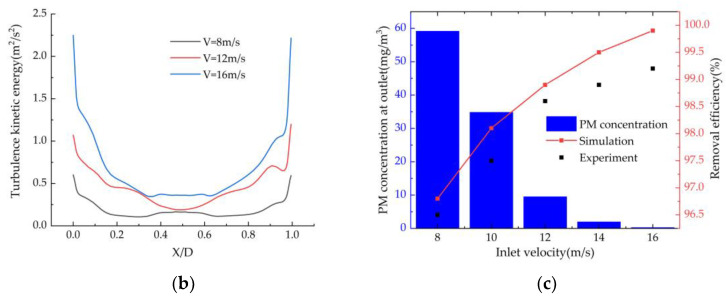
Effect of the gas velocity on the (**a**) particle number density, (**b**) turbulent kinetic energy at Z = 850 mm, and (**c**) particle concentration and removal efficiency.

The changes in the concentration of the fine particles at the outlet when the spray volume is 1.2 L/min, the inlet dust concentration is 2 g/m^3^, and the flue gas flow rate is 8–16 m/s are shown in Figure 6. Figure 6a show that as the airflow rate increases, the number density of the fine particles decreases in each particle size segment after spray agglomeration, but the number of small particles is slightly greater. This indicates that large particles are easily removed, while small particles are relatively difficult to remove. This is due to the fact that the fine particles follow the airflow movement, have a short residence time, and do not make full contact with the droplets.

In order to study the influence of the flow velocity on the turbulent particle coalescence, the distribution curve of the turbulent kinetic energy in transverse Z = 850 mm in the scrubber was attained (Figure 6b). As can be seen from Figure 6b, as the flue gas velocity increases, the Reynolds number in the flow field increases, and the turbulent kinetic energy also increases, which improves the agglomeration efficiency of the particles and promotes the collision and agglomeration of the fine particles and droplets.

As can be seen from Figure 6c, as the air inlet velocity increases from 8 to 16 m/s, the concentration of the particulate matter at the outlet decreases from 60 mg/m^3^ to 2 mg/m^3^, reaching the ultra-low emissions requirement of 5 mg/m^3^. The removal efficiency increases from 96.8% to 99.9% and the fine particle removal efficiency gradually improves and then becomes stable. The efficiency values simulated by the model are within the allowable range of error (about 99–99.5% in dedusting efficiency by experimental data). The reasons for this are as follows. As the gas velocity increases, on the one hand, the dust particle capture efficiency of the droplets increases, the turbulent kinetic energy increases, and the gas–liquid mixing becomes more uniform, the probability of collisions between the droplets and particles increases, and the particle size increases, so more particles are removed under the action of the centrifugal force. 

#### 3.5.2. Effect of Spray Volume on Dust Removal Efficiency

**Figure 7:**
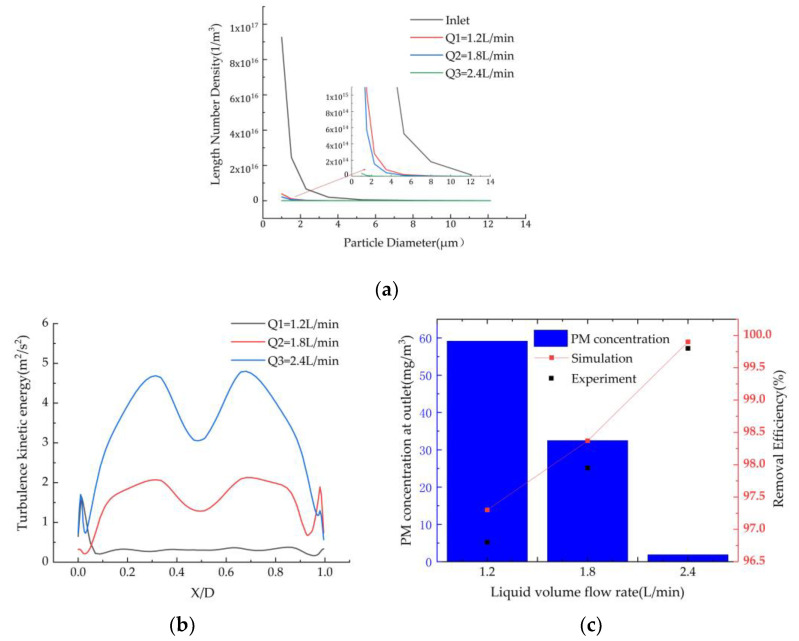
Effect of the spray volumes on the (**a**) particle number density, (**b**) turbulent kinetic energy at Z = 850 mm, and (**c**) particle concentration and removal efficiency.

The variation in the spray volume in the range of 1.2–2.4 L/min was investigated under the conditions that the inlet smoke flow rate was 8 m/s and the concentration was 2 g/m^3^. The number densities of fine particles at the inlet and outlet are shown in Figure 7a. The results show that as the spray volume increases, the number of fine particles in each particle size interval at the outlet decreases, and the rate of decrease of the speed increases. This is because as the volume of the spray increases, the number of droplets increases, and the contact area between the droplets and dust-containing gas also increases, leading to an increase in the probability of the dust particles being captured by the droplets, which can re-enroll the escaped fine particles and dust into the flow field, increase the interaction time between the particles and droplets, and enhance the agglomeration effect.

Figure 7b shows the distribution of the turbulent kinetic energy in transverse Z = 850 mm for different spray flow rates. It can be seen from Figure 7 that as the spray flow increases, the turbulent kinetic energy in the spray area increases, which is conducive to the turbulent mixing of the particles and droplets. In addition, the collision probability and number of particles in a volume unit increase, thus enhancing the turbulent coalescence effect of the particles.

As can be seen from Figure 7c, as the spray volume increases from 1.2 L/min to 2.4 L/min, the collection efficiency increases significantly, and the concentration of the particulate matter at the outlet decreases from 60 mg/m^3^ to 4 mg/m^3^, meeting the ultra-low emission requirement of 5 mg/m^3^. As the spray flow rate increases, the droplet velocity increases, which enhances the inertia effect, diffusion effect, and interception effect of the dust particles, and improves the capture efficiency of the particulate matter. As the flow rate increase, the movement velocity of the droplets increases the disturbance of the surrounding smoke, and the intensity of the turbulence in the spray area increases, which greatly increases the collision probability between the dust particles and droplets. The particle size of the fine particles increases after humidification and agglomeration, and more particles are removed under the action of the centrifugal force. Increasing the slurry spray volume can increase the dust particle removal efficiency, which has been confirmed in many wet dust removal studies [32,33].

#### 3.5.3. Effect of Inlet Particle Concentration on Dust Removal Efficiency

**Figure 8:**
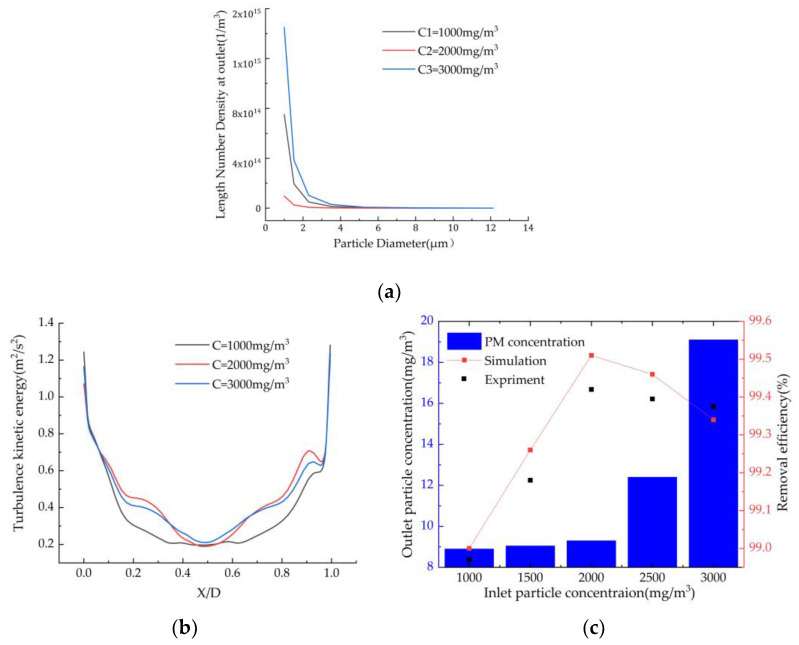
Effect of the particle concentrations on the (**a**) particle number density, (**b**) turbulent kinetic energy at Z = 850 mm, and (**c**) particle concentration and removal efficiency.

The changes in particle concentration in the range of 1–3 g/m^3^ were investigated under the conditions of a flue gas flow rate of 12 m/s and a spray volume of 1.2 L/min. The changes in the number density of the fine particulate matter at the outlet are shown in Figure 8a. As can be seen from Figure 8a, as the particle concentration increases, the number density of the fine particles initially decreases and then increases. Figure 8b shows the turbulent kinetic energy distribution in transverse Z = 850 mm for different particulate matter concentrations. It can be seen from Figure 8b that as the particle concentration increases, the collision kernel function is about the same when the turbulent kinetic energy is about the same.

As can be seen from Figure 8c, as the particulate matter concentration increases, the removal efficiency of the fine particulate matter initially increases and then decreases, and it reaches the highest point at 2 g/m^3^ by the simulation results, which is in good agreement with the experimental results. In addition, the particulate matter concentration at the outlet is less than 10 mg/m^3^. This is because, on the one hand, when the particulate matter concentration is large, the number of particles increases, and the spacing between the particles continuously decreases, which increases the collision probability of the fine particles [20]. This is conducive to enhancing the agglomeration effect of the particles, forming some large particles and thus significantly improving the removal efficiency of the fine particles. On the other hand, because the number of droplets in the scrubber is relatively fixed and the number of dust particles increases, the adsorption efficiency of the spray droplets is limited, which leads to a decrease in the capture efficiency of the dust particles.

## 4. Conclusions

In this study, a mathematical model based on a two-fluid model for cyclonic spray dedusting was developed. The model considers the aggregation between particles and droplets caused by turbulence in detail. The hydrodynamic characteristics in a cyclonic spray scrubber were analyzed and the removal efficiencies were predicted for different gas flow velocities, spray flow rates, and particle concentrations. Based on our analysis, the following conclusions were drawn.

1. The velocity in the cyclonic spray scrubber is basically axisymmetric; that is, the velocity in the central region is the smallest, the velocity gradually increases along the radial direction from the center to the wall, and the velocity gradually decreases to zero near the wall boundary layer, exhibiting the characteristics of swirling flow. The volume concentration distribution of the droplet particles is hollow and conical, the central region is low, and the side wall region is high.
2. As the flue gas flow velocity increases, the turbulent kinetic energy increases, and the efficiency of the turbulent aggregation of the particles increases. The particle size along the axial direction increases, the number density of the fine particles within each particle size interval decreases, and the removal efficiency gradually increases. The particle concentration at the outlet reaches the ultra-low emissions requirement of less than 5 mg/m^3^.
3. As the spray flow rate increases, the number of droplets increases, the contact area between the droplets and air increases, the turbulent kinetic energy in the spray area increases, and the particle size increases significantly after wetting and agglomeration. The particle concentration in all of the size intervals at the outlet decreases, and the removal efficiency reaches 99.7%.
4. As the particle concentration increases, the spacing between the particles decreases continuously, and the particles agglomerate more closely. However, due to the limited adsorption efficiency of the spray droplets, the removal efficiency of the fine particles reaches its highest value at 2 g/m^3^.

## Figures and Tables

**Table 1:** Size distribution of each phase.

FPM Phase		Droplet Phase	
Diameter (μm)	Volume Fraction	Diameter (μm)	Volume Fraction
12.1	0.5	180	0.05
8.0	0.2	142	0.13
5.27	0.11	113	0.35
3.48	0.08	90	0.25
2.29	0.05	71	0.12
1.51	0.035	57	0.08
1.00	0.025	45	0.02

**Table 2:** Comparison of simulation results and experimental data.

Velocity Comparison			Dust Concentration Comparison		
Simulated (m/s)	Measured (m/s)	Error (%)	Simulated (mg/m^3^)	Measured (mg/m^3^)	Error (%)
0.08	0.075	6.25	9.519	9.583	0.67
2.16	2.08	3.70			
6.52	6.34	2.76			
